# Supplementary material for: Predictive modelling of the distribution of Clematis sect. Fruticella s. str. under climate change reveals a range expansion during the Last Glacial Maximum
Source: PeerJ. 2020 Mar 9;8:e8729. doi: 10.7717/peerj.8729 (PMC7067196; doi:10.7717/peerj.8729)
Supplement: Table S2 [file peerj-08-8729-s002.docx]

**Table S2:**

**Proportions of unsuitable areas within the four suitability classes of potential distributions of** ***Clematis* sect. *Fruticella* under four future and three past climate scenarios.**

| Portion of Area | Not Suitable (%) | Low Suitability (%) | Moderate Suitability (%) | High Suitability (%) |
| --- | --- | --- | --- | --- |
| Last inter-glacial | 98.12349633 | 1.472965398 | 0.275676318 | 0.127861957 |
| Last Glacial Maximum | 96.79371025 | 1.435450533 | 1.050561057 | 0.72027816 |
| Mid Holocene | 96.76908659 | 1.637219671 | 1.185556632 | 0.408137105 |
| Current | 96.79233422 | 1.535321187 | 1.122042083 | 0.550302509 |
| ^1^RCP2.6-2050 | 96.93935193 | 1.356147875 | 1.080290501 | 0.624209689 |
| RCP2.6-2070 | 96.81109165 | 1.416801735 | 0.951269784 | 0.820836828 |
| RCP8.5-2050 | 96.78570756 | 1.578991965 | 1.030536231 | 0.604764243 |
| RCP8.5-2070 | 96.9450371 | 1.340577034 | 1.088836358 | 0.625549506 |

^1^ RCP, representative concentration pathway.
