# Supplementary material for: Predictive modelling of the distribution of Clematis sect. Fruticella s. str. under climate change reveals a range expansion during the Last Glacial Maximum
Source: PeerJ. 2020 Mar 9;8:e8729. doi: 10.7717/peerj.8729 (PMC7067196; doi:10.7717/peerj.8729)
Supplement: Table S3 [file peerj-08-8729-s003.docx]

**Supplemental Table S3:**

**Percent contributions and permutation importance of environmental variables included in the Maxent models for Clematis sect. Fruticella under current, past, and future scenarios.**

| **Last Interglacial:** | | |
| --- | --- | --- |
| **Environmental variables** | **Percent contribution** | **Permutation importance** |
| Elevation (GloElev) | 32 | 22.5 |
| Mean UV-B of Lowest Month (UVB4) | 19.1 | 11 |
| Precipitation of Coldest Quarter (BIO19) | 17.8 | 1.6 |
| Mean Temperature of Driest Quarter (BIO9) | 16.1 | 37.1 |
| barren/very sparsely vegetated land (NVG) | 7.1 | 3.8 |
| Mean Diurnal Range (Mean of monthly (max temp - min temp)) (BIO2) | 2.6 | 3.9 |
| Wet days (WET) | 2.3 | 15.1 |
| Precipitation of Warmest Quarter (BIO18) | 2 | 2.6 |
| Precipitation Seasonality (Coefficient of Variation) (BIO15) | 1.1 | 2.2 |
|  |  |  |

| **Last Glacial Maximum:** | | |
| --- | --- | --- |
| **Environmental variables** | **Percent contribution** | **Permutation importance** |
| Elevation (GloElev) | 33.8 | 23 |
| Mean UV-B of Lowest Month (UVB4) | 19.1 | 28.9 |
| Precipitation of Coldest Quarter (BIO19) | 17.8 | 2.5 |
| Mean Temperature of Driest Quarter (BIO9) | 15.5 | 25.6 |
| barren/very sparsely vegetated land (NVG) | 5.8 | 4.1 |
| Precipitation of Warmest Quarter (BIO18) | 2.7 | 1.8 |
| Mean Diurnal Range (Mean of monthly (max temp - min temp)) (BIO2) | 2.4 | 3.5 |
| Wet days (WET) | 1.8 | 9.6 |
| Precipitation Seasonality (Coefficient of Variation) (BIO15) | 1.1 | 1.1 |
|  |  |  |
| **Mid Holocene:** | | |
| **Environmental variables** | **Percent contribution** | **Permutation importance** |
| Elevation (GloElev) | 32 | 22.5 |
| Mean UV-B of Lowest Month (UVB4) | 19.1 | 11 |
| Precipitation of Coldest Quarter (BIO19) | 17.8 | 1.6 |
| Mean Temperature of Driest Quarter (BIO9) | 16.1 | 37.1 |
| barren/very sparsely vegetated land (NVG) | 7.1 | 3.8 |
| Mean Diurnal Range (Mean of monthly (max temp - min temp)) (BIO2) | 2.6 | 3.9 |
| Wet days (WET) | 2.3 | 15.1 |
| Precipitation of Warmest Quarter (BIO18) | 2 | 2.6 |
| Precipitation Seasonality (Coefficient of Variation) (BIO15) | 1.1 | 2.2 |
|  |  |  |
| **Current:** | | |
| **Environmental variables** | **Percent contribution** | **Permutation importance** |
| Elevation (GloElev) | 22.9 | 16.9 |
| Mean UV-B of Lowest Month (UVB4) | 22.2 | 19.2 |
| Precipitation of Coldest Quarter (BIO19) | 20.7 | 1 |
| Mean Temperature of Driest Quarter (BIO9) | 18.5 | 36.6 |
| barren/very sparsely vegetated land (NVG) | 7.5 | 7.4 |
| Mean Diurnal Range (Mean of monthly (max temp - min temp)) (BIO2) | 2.8 | 4.8 |
| Wet days (WET) | 2.2 | 8.9 |
| Precipitation of Warmest Quarter (BIO18) | 2 | 1.7 |
| Precipitation Seasonality (Coefficient of Variation) (BIO15) | 1.1 | 3.5 |
|  |  |  |
| **CCSM4^1^ RCP^2^2.6 2050** | | |
| **Environmental variables** | **Percent contribution** | **Permutation importance** |
| Elevation (GloElev) | 31.4 | 17.7 |
| Mean UV-B of Lowest Month (UVB4) | 19.8 | 23.8 |
| Precipitation of Coldest Quarter (BIO19) | 17.1 | 1.8 |
| Mean Temperature of Driest Quarter (BIO9) | 16 | 29.1 |
| barren/very sparsely vegetated land (NVG) | 6.9 | 5.6 |
| Mean Diurnal Range (Mean of monthly (max temp - min temp)) (BIO2) | 3.4 | 5.6 |
| Precipitation of Warmest Quarter (BIO18) | 2.2 | 1.3 |
| Precipitation Seasonality (Coefficient of Variation) (BIO15) | 1.7 | 2.5 |
| Wet days (WET) | 1.6 | 12.5 |
| **CCSM4 RCP2.6 2070:** | | |
| **Environmental variables** | **Percent contribution** | **Permutation importance** |
| Elevation (GloElev) | 27.7 | 20.2 |
| Mean UV-B of Lowest Month (UVB4) | 21.2 | 17.6 |
| Precipitation of Coldest Quarter (BIO19) | 20.1 | 2.4 |
| Mean Temperature of Driest Quarter (BIO9) | 16.4 | 43.4 |
| barren/very sparsely vegetated land (NVG) | 6 | 3.6 |
| Mean Diurnal Range (Mean of monthly (max temp - min temp)) (BIO2) | 3.2 | 5.4 |
| Precipitation of Warmest Quarter (BIO18) | 3.1 | 2.8 |
| Precipitation Seasonality (Coefficient of Variation) (BIO15) | 1.2 | 1.7 |
| Wet days (WET) | 1 | 2.7 |
|  |  |  |
| **CCSM4 RCP8.5 2050:** | | |
| **Environmental variables** | **Percent contribution** | **Permutation importance** |
| Mean UV-B of Lowest Month (UVB4) | 23.4 | 25.4 |
| Elevation (GloElev) | 23.1 | 16.4 |
| Precipitation of Coldest Quarter (BIO19) | 22.3 | 4.6 |
| Mean Temperature of Driest Quarter (BIO9) | 18.5 | 29.3 |
| barren/very sparsely vegetated land (NVG) | 5.9 | 4.1 |
| Precipitation of Warmest Quarter (BIO18) | 2.3 | 3.6 |
| Mean Diurnal Range (Mean of monthly (max temp - min temp)) (BIO2) | 2.2 | 5.5 |
| Precipitation Seasonality (Coefficient of Variation) (BIO15) | 1.1 | 2.7 |
| Wet days (WET) | 1.1 | 8.4 |
| **CCSM4 RCP8.5 2070:** | | |
| **Environmental variables** | **Percent contribution** | **Permutation importance** |
| Elevation (GloElev) | 24.3 | 16.8 |
| Precipitation of Coldest Quarter (BIO19) | 22.3 | 3.1 |
| Mean UV-B of Lowest Month (UVB4) | 21.2 | 19.7 |
| Mean Temperature of Driest Quarter (BIO9) | 17.5 | 35.6 |
| barren/very sparsely vegetated land (NVG) | 6.8 | 6.6 |
| Precipitation of Warmest Quarter (BIO18) | 3.2 | 1.7 |
| Wet days (WET) | 2 | 11 |
| Mean Diurnal Range (Mean of monthly (max temp - min temp)) (BIO2) | 1.7 | 3.9 |
| Precipitation Seasonality (Coefficient of Variation) (BIO15) | 1 | 1.5 |

^1^CCSM4: Community Climate System Model 4

^2^RCP: Representative Concentration Pathways
